# Adaptation of the Newcastle Disease Virus to Cell Cultures for Enhancing Its Oncolytic Properties

**Published:** 2019

**Authors:** K. S. Yurchenko, Yi. Jing, A. M. Shestopalov

**Affiliations:** Federal State Budget Scientific Institution «Federal Research Center of Fundamental and Translational Medicine», Timakova Str. 2, 630117, Novosibirsk, Russia; Federal State Budgetary Educational Institution of higher professional education “Novosibirsk national research state University», Pirogova Str. 1, 630090, Novosibirsk, Russia

**Keywords:** adaptation, Newcastle disease virus, oncolytic properties, tumor cells, cytotoxic effect

## Abstract

This study focuses on the adaptation of natural Newcastle disease virus (NDV) strains isolated from wild birds to human tumor cells. Many candidates for virotherapy are viruses pathogenic for human. During recombination of genetic material, there always exists a risk of getting a virus with an unstable genome. This problem can be solved by using natural apathogenic viruses as oncolytic agents. The Newcastle disease virus is the causative agent of contagious avian diseases. Its natural strains exhibit an antitumor effect and are considered safe for humans. As shown in earlier studies, the oncolytic properties of natural strains can be enhanced during adaptation to cell cultures, without interference in the virus genome. This study demonstrates that serial passaging increases the viral infectious titer in cancer cells. Moreover, the viability of tumor cells decreases post-infection when Newcastle disease virus strains are adapted to these cell cultures. The findings of this study complement the well-known data on the adaptation of the Newcastle disease virus to human cancer cells. Hence, it is possible to obtain a NDV strain with a more pronounced oncolytic potential during adaptation. This should be taken into account when choosing a strategy for designing anticancer drugs based on this virus.

## INTRODUCTION

Virotherapy is an experimental method of targeted therapy of malignant diseases that employs oncolytic viruses, including the Newcastle disease virus (NDV) (family Paramyxoviridae, genus *Avulavirus*), for selective destruction of tumor cells [1]. Oncolytic viruses selectively infect tumor cells and use their activated synthetic apparatus for replication, which causes a cytotoxic effect (CTE) and leads to cell death [2]. Normal healthy cells remain unharmed due to the development of an early antiviral interferon response, which prevents viral genome replication [3]. The Newcastle disease virus drew researchers’ attention as a potential oncolytic agent in the mid-20th century and has proved to be a safe and effective antitumor agent [4].

NDV strains for virotherapy studies are divided into lytic and non-lytic ones based on the properties of the F protein [5]. Both lytic and non-lytic strains can act as powerful anticancer agents. Lytic strains are advantageous, as infectious viral progeny is produced by multiple replication cycles and the cytotoxic effect spreads in tumor tissue. Non-lytic strains have only one replication cycle. The new virions of non-lytic strains contain inactive variants of the F protein, and the antitumor effects are mainly associated with the stimulation of the immune response [6].

A significant part of virotherapy research focuses on the creation of recombinant strains to enhance the antitumor properties of viruses. However, the development of viral constructs is associated with the need to regulate the biological properties of the recombinant virus, its cancer tropism, and the elimination of possible inertial mutagenesis and recombinations related to the restoration of the properties of viruses that initially were pathogenic for humans [7, 8]. The natural oncolytic potential of NDV strains isolated from wild birds can be used for virotherapy without any complicated modifications of the genome. Different lentogenic (low-pathogenic) and mesogenic (medium-pathogenic) NDV strains have been studied since the 1960s *in vitro*, *in vivo,* and in clinical studies involving cancer patients [9].

Despite the popularity of the genetic engineering methods used to design viruses for virotherapy, natural unmodified strains also can be a powerful antitumor agent. Wild-type NDV strains exhibit a high oncolytic activity. The best studied and clinically tested strains are 73-T, MTH-68, PV701, and NDV-HUJ [10]. These strains belong to the lytic type of virus, which directly kill tumor cells. In addition, virulent mesogenic strains induce a more powerful apoptotic response in tumor cells, in contrast to apatogenic and low-virulent ones [11, 12].

The natural attenuated nonrecombinant strain PV701 obtained from the mesogenic strain MK107 (Gaithersburg, USA) [13] exhibits an antitumor activity against a broad range of tumors of epithelial, neuroectodermal, and mesenchymal origin [10]. Another lytic strain, MTH- 68/H, derived from the natural strain Hertfordshire (Herts’33), exhibits a cytotoxic effect on various human tumor cells [10]. Systemic or locoregional administration of this virus leads to partial or complete regression of primary and metastatic tumors [14, 15]. The adapted natural strain 73-T causes syncytia formation and death of a wide range of malignant cells, both *in vitro *and *in vivo *[16, 17]. Meanwhile, natural mesogenic strains exhibiting a cytotoxic effect on malignant cells are safe for normal human cells [12, 16, 17, 18].

Investigation of the mechanism of viral antitumor cytotoxic action allowed researchers to put forward a hypothesis that a strain with more pronounced lytic properties can be obtained during viral adaptation to a cell culture [19]. During adaptation, variants of the wild-type virus with more effective reproduction in host cells may emerge. On the one hand, there is a risk that the increased infectious potency of the natural virus against adapted cells can reduce its virulence [20, 21]. This may have a negative effect on its oncolytic properties. On the other hand, adaptation of the virus to a specific cell line can increase the cytotoxic potential of the adapted virus against these cells [22].

Thus, the strain based on natural MD20Z (USA, 1945) was obtained by serial (n = 38) passaging in Ehrlich ascites carcinoma cells and was tested on an *in vivo *model. The overall weekly survival rate of mice with ascites tumors after treatment with the late-passage virus increased from 0 to 63.6%. Moreover, the virus actively replicated in tumor cells and exhibited a cytotoxic effect faster than the wild-type strain [23]. Cassel W.A. and Garrett R.E. [24] confirmed that the oncolytic effect is enhanced after adaptation of NDV 379-SI from a natural MD20Z strain by the method of serial (n = 73) passages in Ehrlich ascites carcinoma *in vitro* followed by 13 passages *in vivo*. Later, a number of successful clinical trials of allogeneic and autologous vaccines based on the NDV 73-T strain against metastatic melanoma were conducted in the USA [25].

Therefore, taking into account the data confirming successful adaptation of wild-type strains with an enhanced antitumor effect during sequential passaging, we studied the adaptation of wild-type strains of various pathotypes on lines of human tumor cells in order to verify the effectiveness of the cytotoxic properties acquired after passaging of the strains.

## EXPERIMENTAL

**Cell lines**

We studied transplantable cell lines: Vero – kidney epithelial cells extracted from the African green monkey; HCT116 – human colon carcinoma cell line (State Research Center of Virology and Biotechnology Vector, Novosibirsk region, Russia); Hela – human epithelioid cervix carcinoma (Institute of Clinical and Experimental Lymphology, Novosibirsk, Russia); MCF7 – human breast adenocarcinoma cell line (Institute of Cytology of the Russian Academy of Sciences, St. Petersburg, Russia); and A549 – human non-small lung carcinoma cell line (Research Institute of Fundamental and Clinical Immunology, Novosibirsk, Russia).

Vero and tumor cell lines were grown in Dulbecco’s modified Eagle’s media (DMEM) supplemented with 10% heat-inactivated fetal bovine serum (FBS) and 50 mg/ml gentamicin. Cells were maintained in cultural flasks (25 cm^2^) at 37°C in an incubator with 5% CO_2_.

**Newcastle disease virus strains**

Our study was performed using four NDV strains: two virulent mesogenic NDV strains isolated from rock doves in Russia (NDV/Altai/pigeon/770/2011 [26] and NDV/Altai/pigeon/**777**/2010 [27]) and two lentogenic strains (NDV/mallard/Amur/264/2009 and NDV/ teal/Novosibirsk_region/320/2010) isolated from a mallard and a teal, respectively.

**Preparation of Newcastle disease virus strains**

The viral strains were amplified in the allantoic fluid of 10-day-old embryonated eggs. The presence of the virus was determined by hemagglutination assay (HA) with rooster erythrocytes. The hemagglutinin titers of each viral strain were determined according to the standard procedure recommended by the WHO [23].

**Titration of the Newcastle disease virus on Vero cell line (TCID50)**

Titration of the Newcastle disease virus on the Vero cell line, which is sensitive to the cytopathic effect of NDV, was performed according to the procedure described in [23]. The cell suspension was cultured in the growth medium on 96-well plates at a concentration of 30,000 cells per well under standard conditions.

The next day, the cell monolayer was washed with Hanks’ solution and 10-fold viral dilutions per well were added. The dilutions were prepared using MEM maintenance medium supplemented with 1% heat-inactivated FBS. The plates were incubated under standard conditions for 1 h to adsorb the virus; the virus-containing liquid (VCL) was then replaced with the maintenance medium. Every day, the cell monolayer was inspected under a microscope to detect the presence of CTE. The final titration was carried out on day 4. The infectious titers of the virus on the Vero cell line were calculated according to the Cerberus method in the Ashmarin’s modification and presented as lgTCID50/ml [30].

**Serial passages of NDV strains in transplantable Vero cell line and tumor human cell lines**

Preparation of the cells and infection with 10-fold dilutions of the virus were performed using the same procedure as the one described in the titration method. On day 4 post-infection, the infectious titer was counted and VCL of the extreme dilutions was collected. The next passage was carried out with infection of Vero cells with the virus collected in extreme dilution using the same method. Hence, we conducted a total of eleven passages and calculated the infectious titer of each passage.

**Colorimetric analysis of cell viability after infection with NDV strains (the MTT assay)**

The viability of human tumor cell lines was analyzed using the MTT assay 4 days post-infection with viral strains. The one-day-old monolayer of tumor cells on the 96-well plate was washed with Hanks’ solution and incubated with viruses (titer 8 HAU per 10,000 cells) for 1 h under standard conditions. The cells were then washed with Hanks’ solution and incubated in 200 μl of the maintenance medium for 4 days under standard conditions. Control tumor cells without the virus were incubated in MEM maintenance medium.

On day 4, the cells were washed with Hanks’ solution. The MTT working solution at a concentration of 5 mg/ml was prepared using the MEM maintenance medium. The MTT solution (100 μl) was added to each well. The plate was incubated for 4 h under standard conditions. The MTT solution was then replaced with DMSO (150 μl per well), and the cells were incubated for 1 h in the dark at room temperature. The optical density was measured at wavelengths of 540 nm and 630 nm on a LonzaBiotek ELX808 absorbance microplate reader (USA). Cell viability was evaluated according to the ratio between the relative percentage of living and control uninfected cells using the formula: (V_540_-V_630_)/(K_540_-K_630_) × 100%, where V and K denote the values for virus-infected and control wells, respectively.

**Infection of human tumor cell lines with mesogenic NDV strains to assess the replicative activity of the virus in different tumor cells**

The replicative activity of NDV strains in different tumor cell lines was assessed by titration on HCT116, HeLa, A549, and MCF7 cells after infection with NDV strains.

Human tumor cell lines and control Vero cells were cultured in a growth medium in culture flasks (25 cm^2^) under standard conditions. The one-day-old cell monolayer was washed twice with Hanks’ solution and incubated with viruses in a 1-ml volume (titer 8 HAU per 10,000 cells) with adsorption for 45 min under standard conditions. The cells were then washed twice with Hanks’ solution, and a fresh 10 ml of the MEM maintenance medium was added. The flasks were incubated under standard conditions. The infectious titer of the culture medium was determined 3, 6, 24, 48, and 72 h post-infection with NDV strains by titration on the respective tumor and model cell lines using the conventional method.

**Statistical analysis**

Statistical data were obtained using the Statistica 6.0 software. The Student’s t-test was used to determine the statistical significance for comparing the individual data points.

## RESULTS AND DISCUSSION

Newcastle disease virus strains were propagated in the allantoic fluid of 10-day-old embryonated eggs. The presence of the virus in allantoic fluid was confirmed by hemagglutination assay with rooster erythrocytes; infectious viral titer was determined in the Vero cell line (*Table 1*). A high titer of the mesogenic strains isolated from rock doves was shown in hemagglutination assay and during titration on Vero cells.

**Table 1:** Titers of wild-type Newcastle disease virus strains

Strain	HA assay/50 μl	lgTCID50/ml
NDV/Altai/ pigeon/770/2011	512	6.7
NDV/Altai/ pigeon/777/2010	256	6.5
NDV/mallard/ Amur/264/2009	64	3.0
NDV/teal/Novosibirsk_ region/320/2010	64	3.0

Two possible systems for enhancing the antitumor properties of the Newcastle disease virus were used in the next step. The first system consisted in passaging NDV strains in transplantable sensitive Vero cells with viral adaptation to this line, followed by testing of the viability of tumor cells after infection with adapted NDV strains. Vero cells do not synthesize IFNα and IFNβ, which allows the Newcastle disease virus to reproduce freely, release new viral progeny, and form CTE.

**Fig. 1:**
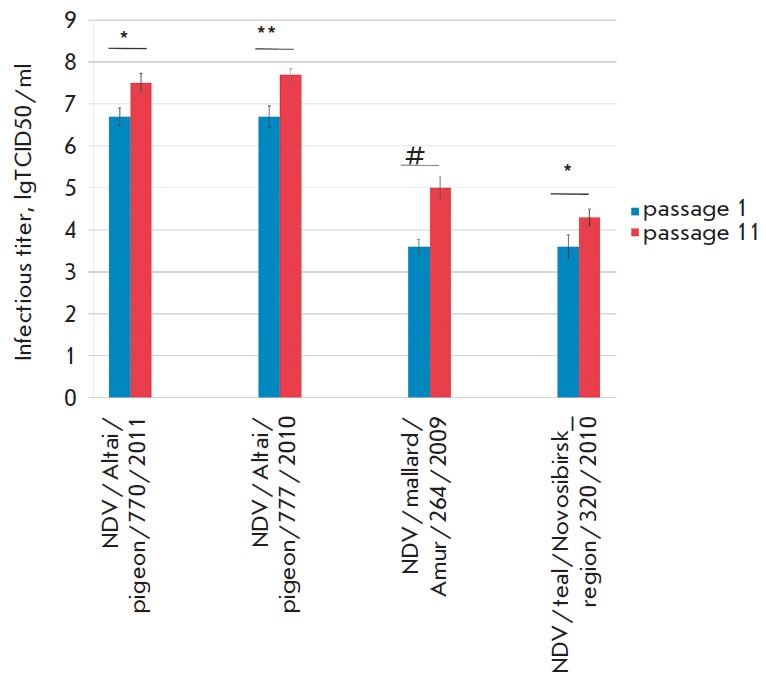
The increasing infectious titers of Newcastle disease virus strains during the adaptation to the Vero cell culture. * *p *= 0.05; ** *p *= 0.01; # *p *= 0.001 (Student’s t-test)

The second system consisted in the adaptation of strains to tumor cell lines with assessment of the dynamics of changes in the viability of tumor cells after infection with adapted strains in late passages.

**Table 2:** The dynamics of titer changes for Newcastle disease virus strains during adaptation to the Vero cell line: mean relative value ± standard deviation

NDV strains	Passage										
	1	2	3	4	5	6	7	8	9	10	11
NDV/Altai/pigeon/770/2011	6.7±0.21	6.8±0.52	6.7±0.39	6.9±0.15	6.9±0.18	6.9±0.23	7.0±0.25	7.5±0.31	7.5±0.14	7.5±0.34	7.5±0.24
NDV/Altai/pigeon/777/2010	6.7±0.26	6.7±0.28	6.9±0.31	6.9±0.21	7.0±0.19	7.0±0.23	7.2±0.33	7.7±0.23	7.7±0.21	7.7±0.40	7.7±0.14
NDV/mallard/Amur/264/2009	3.6±0.18	3.6±0.19	3.8±0.22	3.8±0.25	4.5±0.24	4.7±0.36	4.7±0.33	5.0±0.40	5.1±0.36	5.0±0.22	5.0±0.28
NDV/teal/Novosibirsk_region/320/2010	3.6±0.28	3.6±0.21	3.8±0.18	3.7±0.19	4.3±0.31	4.2±0.14	4.3±0.29	4.3±0.18	4.3±0.26	4.4±0.33	4.3±0.20

According to the first scheme, the viral titers of the mesogenic strains NDV/Altai/pigeon/770/2011 and NDV/Altai/pigeon/777/2010 increased to 7.5 and 7.7 lgTCID50/ml, respectively (*Table 2*, *Fig. 1*) after 11 passages in the Vero cell line. The titer of these strains was initially higher than those of lentogenic strains and was 6.7 lgTCID50/ml after the first passage. We failed to reach values as high as the titers of the lentogenic strains NDV/mallard/Amur/264/2009 and NDV/teal/Novosibirsk_region/320/2010 for mesogenic strains in late passages, despite the fact that the titers increased from 3.6 to 5.0 lgTCID50/ml and from 3.6 to 4.3 lgTCID50/ml, respectively. The presence of a “virulent” sequence at the F protein cleavage site is probably affected not only by the virus’ ability to efficiently replicate in cells with the formation of CTE. It is quite possible that belonging to a certain pathotype, which is directly associated with the cytolytic capacity, also plays a role, since all the studied strains contained a “virulent” sequence at the cleavage site, but only the NDV/Altai/pigeon/770/2011 and NDV/Altai/pigeon/777/2010 belonged to the mesogenic type [26, 27].

**Fig. 2:**
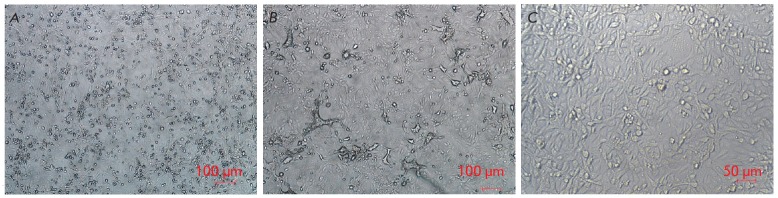
The cytotoxic effect of the NDV/Altai/pigeon/770/2011 strain on a Vero cell culture, 7^th^ passage on cells. *A *– cells infected on day 3 after incubation with the virus; *B *– cells infected on day 4 after incubation with the virus; *C *– syncytium formation in the infected cell culture on day 2 after incubation with the virus

During adaptation to the Vero cell line, the titer of all studied viral strains increased and remained unchanged after seven or eight passages. In addition to the increase in infectious titer in the TCID50 assay, the morphological changes in the cell monolayer became more pronounced. After infection with mesogenic strains, a cytotoxic effect was observed in the 1^st^–3^rd^ passages in Vero cells on day 2 or 3 of cultivation. Areas of rounded dead cells formed in the culture: they were detached from the plate surface and spread in the monolayer by day 4. In the next 5–11 passages, bundles consisting of cell agglomerates formed in the monolayer on days 3–4, in addition to the dead rounded cells (the standard manifestation of the cytotoxic effect). Syncytia-like structures appeared by the 7^th^–10^th^ passages (on days 2–3), which are characteristic of CTE upon infection with paramyxoviruses (*Fig. 2*). Despite the difference in CTE in different passages, the maximum lesion of the monolayer amounted to about 90%, regardless of passage; the destructive changes were usually most pronounced on day 4 post-infection.

**Fig. 3:**
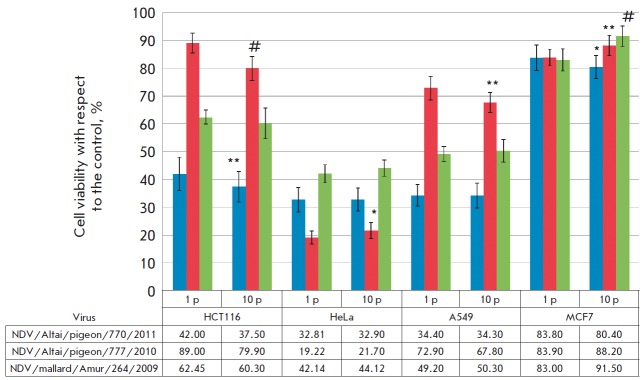
Comparative assessment of the viability of human tumor cell cultures depending on infection with NDV strains of the 1^st^ (1 p) and 10^th^ (10 p) passages in a Vero cell line. MTT assay, * *p *= 0.05; ** *p *= 0.01; # *p *= 0.001 (Student’s t-test)

Syncytia formation, which is typical in cultivation of different paramyxoviruses, took place only in a few cases: the NDV/Altai/pigeon/770/2011 strain in the 7^th^ passage in Vero cells (*Fig. 2B*) and the NDV/mallard/ Amur/264/2009 strain in the 10^th^ passage on days 2 and 3, respectively. Syncytia formation was not observed during primary passaging of these viruses in Vero cells.

No morphological changes in the monolayer were observed during passaging of the NDV/mallard/ Amur/264/2009 and NDV/teal/Novosibirsk_region/320/2010 strains, except for a single case of syncytium formation in the monolayer.

The adapted mesogenic strains NDV/Altai/pigeon/770/2011, NDV/Altai/pigeon/777/2010, and lentogenic strain NDV/mallard/Amur/264/2009 in the 10^th^ passage were used to perform a comparative evaluation of their anticancer properties against tumor cell lines (HCT116, HeLa, A549, and MCF7).

The multiplicity of infection of tumor cell lines was 8 HAU/10,000 cells. Cell survival after infection was assessed by MTT assay on day 4 (*Fig. 3A*).

A comparison of the viability of tumor cell lines infected with viral strains of the 1^st^ and 10^th^ passages in Vero cells did not reveal any significant increase in the antitumor activity of NDV. The HCT116 cell line was an exception: infection with NDV/Altai/pigeon/770/2011 and NDV/Altai/pigeon/777/2010 of the 10^th^ passage in Vero cells reduced the viability of this cell line by 5.0 and 9.9%, respectively.

**Fig. 4:**
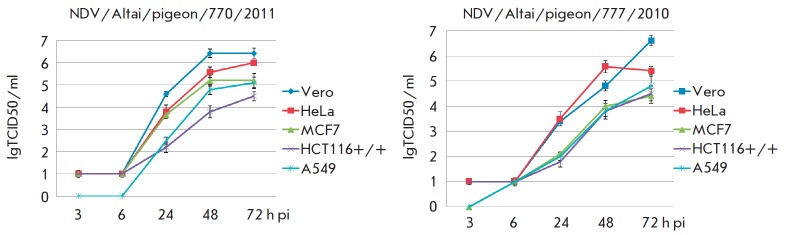
The dynamics of the replicative activity of Newcastle disease virus strains in a Vero cell culture and human tumor cell cultures, h pi – hours post-infection

In the next step of this study, the strains were adapted to each tumor cell line in order to check whether their antitumor potential increases upon adaptation to a specific tumor cell line or not.

**Table 3:** The dynamics of titer changes for the NDV/Altai/pigeon/770/2011 strain during adaptation to tumor cell lines: mean relative value ± standard deviation

NDV strains	Passage										
	1	2	3	4	5	6	7	8	9	10	11
HCT116	4.5±0.33	4.6±0.32	4.6±0.47	4.6±0.40	5.0±0.60	5.4±0.28	5.2±0.39	5.6±0.41	6.0±0.14	6.4±0.63	6.4±0.56
HeLa	6.0±0.54	5.8±0.44	5.6±0.61	5.8±0.56	5.8±0.36	5.4±0.42	6.0±0.55	6.0±0.61	6.2±0.21	6.8±0.61	6.6±0.78
A549	5.1±0.58	5.4±0.41	5.2±0.37	5.6±0.26	5.8±0.33	5.8±0.29	5.8±0.34	5.8±0.36	6.2±0.36	6.0±0.52	6.0±0.45
MCF7	5.2±0.43	5.2±0.43	5.2±0.51	5.0±0.44	5.6±0.49	5.8±0.52	5.8±0.55	5.8±0.32	5.8±0.26	5.8±0.48	5.8±0.36

The dynamics of the replication activity of mesogenic strains was determined for all tumor cell lines and for the Vero cell line as a positive control (*Fig. 4*). Titration of VCL of NDV/Altai/pigeon/770/2011 collected after 3, 6, 24, 48 and 72 h showed the dynamics of viral titer growth. The highest titer on day 3 was observed for Vero cells; the lowest titer, for the HCT116 cell line. Similar data were obtained for the NDV/Altai/ pigeon/777/2010 strain. The lowest titer was obtained on the HCT116 and MCF 7 cell lines.

**Fig. 5:**
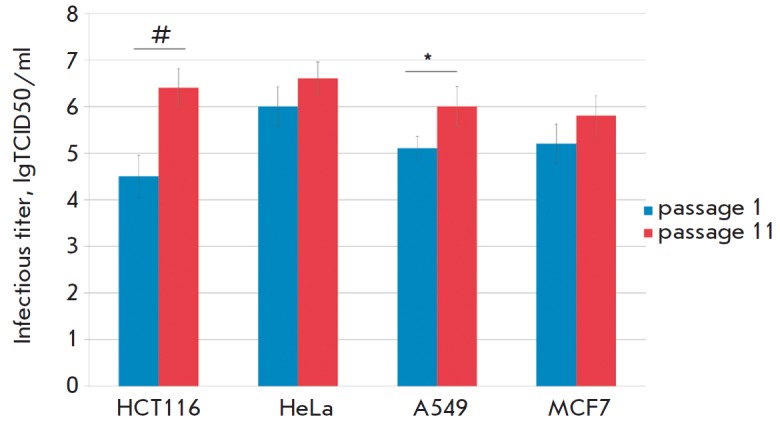
The increasing infectious titers of Newcastle disease virus strains during adaptation to human tumor cell cultures, * *p *= 0.05; # *p* = 0.001 (Student’s t-test)

The NDV/Altai/pigeon/770/2011 and NDV/Altai/ pigeon/777/2010 strains are similar in their characteristics and replication activity, so one strain, NDV/ Altai/pigeon/770/2011, was selected for further study focused on virus adaptation to tumor cell lines with a view to increasing the oncolytic potential. This strain was cultured on tumor cell lines until the 11th passage as described above (*Table 3*, *Fig. 5*).

**Fig. 6:**
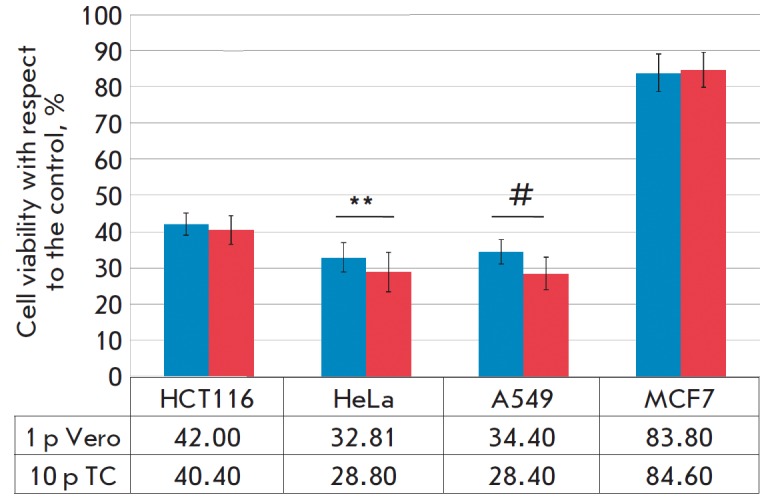
Comparative assessment of the viability of human tumor cell cultures depending on infection with NDV/ Altai/pigeon/770/2011 strain of the 1^st^ passage (1 p) in Vero cells and 10^th^ passage (10 p) in tumor cells (TC), MTT assay. ** *p *= 0.01; # *p *= 0.001 (Student’s t-test)

The titer increased after virus passage (passages 1–11) in the cell lines: in HCT116, by 1.9 lgTCID50/ ml; in HeLa, by 0.6 lgTCID50/ml; in A549, by 0.9 lgTCID50/ml; and in MCF7, by 0.6 lgTCID50/ml. However, an increase in viral titer by almost two orders of magnitude was observed only in viral adaptation to HCT116 cells. The viability of tumor cell lines after infection with 10^th^ passage strains decreased by 1.6, 4.1, and 6.0% in HCT116 HeLa, and A549 cells, respectively, and increased by 0.8% in MCF7 cells (*Fig. 6*).

A different technique might be required for the adaptation to tumor cells, which would include culturing in higher passages or passaging in the allantoic fluid of ECE and in its combination with ECE *in vitro*.

Therefore, there probably is no direct correlation between the dynamics of virus development in tumor cell lines and the effectiveness of antitumor action via direct oncolysis of these cells. This fact can be used to confirm the differences in the sensitivity of each specific cell line to the oncolytic activity of the virus. Thus, we managed to reduce the viability of HCT116 cells by 5 and 10% after they had been infected with the NDV/Altai/pigeon/770/2011 and NDV/Altai/pigeon/777/2010 strains, which were passaged in transplantable Vero cells. The HCT116 cell line is perhaps more sensitive to infection with adapted strains. We assume that this effect can be enhanced by longer passaging of strains (probably, in combination with passaging in the allantoic fluid of ECE).

## CONCLUSION

Virus adaptation can be employed as a method to increase the infectious titer in order to subsequently use the virus as an antitumor agent exhibiting more pronounced oncolytic properties. The sensitive Vero cell line can be used to develop wild-type NDV strains. Adaptation of NDV strains to Vero cells (11 passages) statistically significantly increases the viral titer regardless of virulence and initial titer: the average infectious titer was increased by 0.8 and 1.1 lgTCID50/ml for mesogenic strains and by 0.7 and 1.4 lgTCID50/ml for lentogenic strains.

The viability of tumor cells sensitive to NDV in the 1^st^ passage decreased after infection with the adapted strain NDV/Altai/pigeon/770/2011 in the 11th passage. MCF7 cells remained insusceptible to both NDV in the 1^st^ and 11th passages. This result indicates that it is possible to enhance oncolytic properties by strains adaptation and optimization of the passaging procedure.

## Abbreviations

NDV: Newcastle disease virus
DMSO: dimethyl sulfoxide
IFN: interferon
ECE: embryonated chicken eggs
TCID50: 50% tissue culture infective dose
FBS: fetal bovine serum
CTE: cytotoxic effect
VCL: virus-containing liquid
